# Questions of Maternal Influence

**Published:** 1878-11

**Authors:** 


Questions of Maternal Influence.—I take from the Michigan *Medical News* the following extract, which I do not think conveys the meaning I intended it should, and, as a consequence, does me injustice. "The *Medical and Surgical Reporter* says: 'Financial doctors say the cause of the hard times is lack of confidence.' This was not the matter with a husband down South, whom Dr. R. L. Payne tells about in his address as President of the North Carolina Medical Association. He was speaking of 'mother's marks' and gave this example: 'A black child was born to a white married woman in my county, and she accounted to her husband for its very dusky hue by assuring him that she had been terribly frightened by a negro man, who had presented himself before her in a half nude state. The husband was satisfied, and is still happy.'"

The quotation is probably correct, but I certainly intended to have written the last sentence thus: *The husband was satisfied, and is still happy! !* And as the example was given immediately after quoting the case of Maria Theresa, I intended to convey the idea that although I was a firm believer in "mother's marks," there might possibly be another way of accounting for the *color* in both instances, as well as in the case in Virginia, in which the child was *black because the mother ate black walnuts!*—R. L. PAYNE, M.D., *in Med. and Surg. Reporter.*

